# Potential antivirulence activity of sub-inhibitory concentrations of ciprofloxacin against *Proteus mirabilis* isolates: an in-vitro and in-vivo study

**DOI:** 10.1186/s12941-024-00704-4

**Published:** 2024-05-27

**Authors:** Mohamed A. Elhosseini, Tarek E. El-Banna, Fatma I. Sonbol, Maisra M. El-Bouseary

**Affiliations:** 1 Department of Microbiology and Immunology, Faculty of Pharmacy, Tanta University, Tanta, Egypt; 2 Specialized Medical Hospital, Mansoura University, Mansoura, Egypt

**Keywords:** Biofilm, Ciprofloxacin, *Proteus mirabilis*, Sub-inhibitory concentration, Virulence

## Abstract

**Background:**

*Proteus mirabilis* is a significant nosocomial pathogen that is frequently associated with a wide range of infections, necessitating heightened attention to mitigate potential health risks. Hence, this study was performed to investigate the impact of sub-minimum inhibitory concentrations (MICs) of ciprofloxacin (CIP) on *Proteus mirabilis* clinical isolates.

**Methods:**

The sub-MICs of CIP were selected using the growth curve approach. The untreated and treated isolates with sub-MICs of CIP were assessed for their biofilm development, motilities on agar, and other virulence factors. The cell morphology of untreated and treated isolates with sub-MIC of CIP was explored using electron microscope. Moreover, the expression levels of the virulence genes in isolates were measured using quantitative real-time PCR.

**Results:**

Data revealed that sub-MICs of CIP significantly (p < 0.05), in a concentration-dependent manner, inhibited biofilm formation and other virulence factors in the selected isolates. Electron microscope analysis showed cell enlargement and various abnormalities in the cell wall and membrane integrity.

**Conclusion:**

Sub-MICs of CIP exhibited inhibition of virulence and alterations in morphological integrity against *P. mirabilis* isolates.

## Background

*Proteus mirabilis* is a Gram-negative bacterium that belongs to the family *Enterobacteriaceae*. It is well-known for its urease production and distinctive ability to swarm, which looks like a bull's-eye on agar plates [1]. It is an opportunistic pathogen that can cause a wide range of infections in humans, including those of the wounds, the eyes, neonatal meningoencephalitis, empyema, osteomyelitis, respiratory system, and gastrointestinal tract, though it is most commonly associated with urinary tract infections. [2]. The pathogenicity of *P. mirabilis* is mainly related to virulence factors such as fimbriae, flagella, toxins like hemolysin, and extracellular enzymes like urease and protease, in addition to crystalline biofilm formation [3].

Ciprofloxacin (CIP) is a renowned second-generation, broad-spectrum fluoroquinolone antibiotic with potent bactericidal activity against clinically important bacteria that cause a range of diseases, particularly UTIs [4]. Moreover, CIP has better bioavailability as well as enhanced pharmacokinetic and pharmacodynamic properties [5]. Indeed, the World Health Organization has designated ciprofloxacin as a critically important antibiotic [6].

Antibiotics are still the most effective means of controlling bacterial pathogens. To exert their effect successfully, antibiotics should be taken in successive doses at a concentration above the minimum inhibitory concentration (MIC) [7]. However, they only exceed the MIC during treatment for a certain period, after which it transiently falls below the MIC because of the pharmacokinetics of the antibiotics until the subsequent administration restarts the cycle [8]. Additionally, bacteria may encounter sub-MICs due to the use of antibiotics in livestock farming and agriculture, drug-drug interactions, an unlikely dose regimen, and certain clinical or health conditions of the patient that may decrease the bioavailability of antibiotics [9].

Antibiotics at these concentrations (sub-MICs) are not lethal, but they can affect bacteria in a variety of ways, including affecting biofilm formation, inducing morphological changes, influencing the cell surface structure, impacting motility as well as enzyme and toxin production, and altering bacterial adhesion to host cells [10]. Therefore, it is of clinical significance to expand our current understanding of the effects of sub-MICs of antibiotics. The aim of this study was to explore how sub-MICs of CIP affect *P. mirabilis* virulence, fine and ultrastructure, motility, and biofilm formation.

## Materials and methods

### Isolation and preservation of *P. mirabilis*

Clinical isolates were collected from patients admitted to different departments of Tanta University Hospitals over a period from November 2020 to September 2021. The identification of the isolates was conducted through microscopical and routine biochemical techniques [11]. *Proteus mirabilis* ATCC 35659 was used as a reference strain. The isolates with confirmed identity were preserved at – 70 °C in a Luria–Bertani (LB) broth with 25% v/v glycerol [11].

### Antimicrobial susceptibility testing

Clinical isolates susceptibility pattern to various antibiotics was determined using the Kirby-Bauer disc diffusion method [12], and the results were interpreted in accordance with the guidelines of the Clinical and Laboratory Standards Institute (CLSI) [13]. *Escherichia coli* ATCC 25922 was used as the control strain. The antibiotic disks used (Oxoid, UK) were gentamicin, tobramycin, piperacillin/tazobactam, imipenem, cephazolin, cefoxitin, cefotaxime, cefepime, ciprofloxacin, trimethoprim/sulfamethoxazole, aztreonam, ampicillin, amoxycillin/clavulanic acid, and doxycycline.

### Phenotypic screening of virulence factors

Virulence determinants of isolates were screened for the identification of the most virulent strains showing strong biofilm formation and positive for urease, protease, and hemolysin production as follows:

#### Biofilm production

The isolates were cultured in 96-well plates containing tryptic soy broth (TSB) and 1% glucose for 24 h at 37 °C. A negative control containing TSB only was also performed. The plates were washed twice with phosphate-buffered saline (PBS) after discarding the media. The resulting biofilms were fixed with 150 μl of methanol for 20 min, then stained for 15 min with 0.1% crystal violet, followed by rinsing with PBS. The residual biofilms were solubilized using 33% (v/v) glacial acetic acid. The optical density (OD) was measured at 570 nm by a microplate reader (Sunrise Tecan, Grodig, Austria) [14]. Isolates were then classified as negative, weak, moderate, or strong biofilm producers [15].

#### Enzymes and toxin production

The isolates were inoculated into urea broth for urease detection and incubated at 37 °C for 24 h until a color change to magenta (pink) could be observed [16]. Protease was detected on Mueller–Hinton agar containing 3% skimmed milk. After 48 h of incubation at 37 °C, plates were examined to evaluate the formation of lysis zones around the inoculated bacteria [16]. Hemolysin was detected on Columbia blood agar plates. After incubation at 37 °C for 48 h, the zone of hemolysis could be observed [16].

### Determination of minimum inhibitory concentration (MIC)

The agar dilution assay based on CLSI guidelines [12, 13] was used to determine the MIC values of ciprofloxacin (CIP) against *P. mirabilis* isolates. *E. coli* ATCC 25922 was used as the control strain.

### Growth curve assay

The sub-MICs of CIP for all further experiments were selected by plotting the growth curve using a spectrophotometric method [17]. Briefly, isolates were cultured in the absence or presence of CIP (at 1/2, 1/4, or 1/8 MICs) at 37 °C. At specific time intervals, an aliquot was collected from each culture, and the absorbance was read at 600 nm.

### Biofilm assay

The impact of CIP was evaluated against biofilm-producing *P. mirabilis* isolates using a crystal violet assay, as described previously [14]. CIP was introduced to TSB containing bacteria of 10^6^ CFU/ml at sub-MICs (1/4 or 1/8 MICs), and culture without adding any antibiotics was used as a control [18]. The formula used to determine the percent reduction in biofilm formation was:

$$\left[({\text{Control OD}}_{570\,{\text{nm}}} - {\text{Treated OD}}_{570 \,{\text{nm}}})/{\text{Control OD}}_{570\,{\text{nm}}}\right] \times 100.$$

### Motility assay

For the swarming assay, a 0.5 McFarland solution of *P. mirabilis* overnight culture was centrally inoculated on dried LB agar (1.5%) plates in the absence or presence of CIP (at 1/4 or 1/8 MICs). The plates were incubated overnight at 37 °C, and the swarming zones were measured in mm [19]. Similarily, the isolates were stabbed into the center of the dried LB agar (0.4%) plates with or without CIP (at 1/4 or 1/8 MICs). The plates were incubated overnight at 37 °C, and the swimming zones were measured in mm [19].

### Urease assay

The isolates were cultured in LB broth overnight at 37 °C with or without CIP (at 1/4 or 1/8 MICs). Following that, the isolates were centrifuged, re-suspended in PBS, and adjusted to 1.0 at OD_600_. Then, 100 µl of each bacterial suspension was inoculated into a test tube containing 9.5 ml of Christensen’s medium in liquid form and 0.5 ml of urea (at 40%) and incubated at 37 °C for 3 h. After incubation, bacterial cells were centrifuged, and the change in the color of the supernatant was measured at 570 nm [20]. The percent reduction in enzyme production was calculated as previously mentioned.

### Protease assay

The isolates were cultured overnight at 37 °C in LB broth with or without CIP (at 1/4 or 1/8 MICs). The culture supernatant (1 ml) was mixed with an equal volume of 1% (w/v) casein in 0.1 M sodium phosphate buffer (pH 7.0). The mixture was then incubated for 10 min at 30 °C before being terminated with the addition of 2 ml of 0.4 M trichloroacetic (TCA) acid. The mixture was then incubated at room temperature for 30 min before being centrifuged at 10,000 rpm for 5 min. The supernatant (1 ml) was then mixed with 5 ml of 0.4 M Na_2_CO_3_ for 10 min, and then 1 ml of Folin’s reagent mixed with 3 ml of distilled water (1:3 v/v) was added. The mixture was left to stand at 30 °C for 30 min before the OD at 660 nm was measured [21]. The percent reduction in enzyme production was calculated as previously mentioned.

### Hemolysin assay

Isolates were grown overnight at 37 °C in LB broth with or without CIP (at 1/4 or 1/8 MICs). Following centrifugation and filtration of the supernatant, 600 µl was combined with 600 µl of a 2% suspension of red blood cells (RBCs) in saline and incubated at 37 °C for 2 h. The suspension was then centrifuged for 8 min at 4 °C at 10,000 rpm, and the released hemoglobin was determined by measuring absorbance at 540 nm. In the same conditions, a negative control of erythrocytes in LB broth and a positive control of totally hemolyzed erythrocytes by adding sodium dodecyl sulfate (0.1%) were employed [22]. The percentage of hemolysis inhibition was calculated as follows:

$$\left[({\text{sample OD}}-{\text{negative control OD}})/ ({\text{positive control OD}}-{\text{negative control OD}})\right] \times 100.$$

### Fine and ultrastructure investigation

A representative isolate (code P17) was cultured overnight at 37 °C in LB broth with and without a sub-MIC of CIP. Then, the solutions (treated and control) were washed with PBS three times, and the final pellets were processed for further examination. The final pellets were resuspended, fixed with 2.5% glutaraldehyde in PBS buffer (pH 7.4) for 2 h at room temperature, and then postfixed with 1% OsO_4_ in PBS buffer (pH 7.4) for 1 h at 4 °C. A droplet of each bacterial suspension was placed on the microscope slide, then dehydrated with ethanol, and air-dried. The slides were then mounted on metal stubs and sputter-coated with gold, then examined using a scanning electron microscope (SEM) (Akashi Seisakusho, Japan) [23]. The final pellets were fixed for 24 h in 2.5% glutaraldehyde in PBS (pH 7.4), rinsed with the same buffer, and then postfixed for 2 h in 1% OsO_4_ in PBS (pH 7.4). The samples were then dehydrated with ethanol and embedded in Epon 812 epoxy resin. Ultrathin sections were cut on an ultramicrotome, which were then double stained with uranyl acetate and Reynolds lead citrate, then examined using a transmission electron microscope (TEM) (Jeol-1200 ECII, Japan) [23].

### Relative gene expression analysis

The total RNA was extracted from untreated and ciprofloxacin-subMIC-treated *P. mirabilis* isolates using the PureLink^®^ RNA Mini Kit (Thermo Scientific, USA). The yield and purity of the RNA extracted were measured using a NanoDrop spectrophotometer (ThermoFisher Scientific, USA). Then, RNA was reverse transcribed into cDNA using power cDNA synthesis kit (iNtRON Biotechnology, Korea). To analyze the expression pattern of selected genes in the absence and presence of sub-MIC of ciprofloxacin, qPCR was carried out using Power SYBR^®^ Green PCR Master Mix (ThermoFisher Scientific, USA) in Rotor-Gene Q (Qiagen, USA). The expression of selected genes using specific primers (Table 1) was normalized by the housekeeping 16s rRNA gene, and the relative gene expression was determined by the 2^−ΔΔCt^ method [24]. A PCR mixture without a template was used as a negative control. Only genes with a relative 2^−ΔΔCt^ value above 1.0 or below 1.0 were considered significant [25].

**Table 1:** Primers used in real-time PCR

Genes	Primers (5′–3′)	References
*16s rRNA*	F: CCAGACTCCTACGGGAGGCAG R: CGTATTACCGCGGCTGCTG	[26]
*flhDC*	F: CCGCAATGTTTAGACTGGGT R: TTGCAAATCATCCACTCTGG	[26]
*rsmA*	F: TCGAGTTGGTGAAACGCTTA R: TGAGTTTTCTCGGCCTGAAT	[26]
*mrpA*	F: ACACCTGCCCATATGGAAGATACTGGTACA R: AAGTGATGAAGCTTAGTGATGGTGATGGTGATGAGAGT AAGTCACC	[27]
*ureC*	F: TGGCAAGGCAGGTAATCCAG R: ATTGGGCTCTCCTACCGACT	[28]
*zapA*	F: TGGCGCAAATACGACTACCA R: TATCGTCTCCTTCGCCTCCA	[28]
*hmpA*	F: GTTGAGGGGCGTTATCAAGAGTC R: GATAACTGTTTTGCCCTTTTGTGC	[29]

### In-vivo wound infection model

The Research Ethics Committee (Faculty of Pharmacy, Tanta University, Egypt) approved the following protocols while they followed the standard rules for handling and caring for laboratory animals (TP/RE/2/24 p-01). The procedures were established as previously performed [30]. Male BALB/c mice (n = 30) were obtained from the Cairo University College of Veterinary Medicine's animal house (Cairo, Egypt). Mice weighed 120–150 g and were 6–8 weeks old at the time of the study. Following the creation of the wound infection, all mice were housed as individuals in a ventilated cage to prevent fighting and cross-contamination. They were supplied with free access to food and filtered water and were kept on a 12 h light/dark cycle at room temperature. Three groups of mice (10 mice each) were assigned at random. The control group, Group I, was not treated with any materials. Groups II and III were given bacteria and treated with 1/4 or 1/8 MICs of CIP, respectively. Under xylazine (5 mg/kg) and ketamine (40 mg/kg) anaesthesia, the backs of all mice were shaved and sterilized with 10% povidone-iodine. A nearly 10-mm circular incision was made on the dorsal inter-scapular region of each animal. The wounds were infected with 10 μl of the bacterial suspension in PBS (10^6^ CFU). Following 30 min of inoculation, 20 μl of the vehicle (PBS) was injected subcutaneously in the control group I, and CIP (at 1/4 or 1/8 MICs) was injected subcutaneously in the treated groups II and III, respectively.

On the sixth day of the experiment, mice were anesthetized with isoflurane and were euthanized by cervical dislocation (CD) according to the American Veterinary Medical Association (AVMA) Guidelines for the Euthanasia of Animals (2020 Edition). Hematoxylin–eosin (H&E) staining was employed for histopathological examination of the skin lesions.

### Statistical analysis

A one-way ANOVA was employed for comparison. A p-value < 0.05 was considered statistically significant. GraphPad Prism version 5 software was used to create graphs and run statistical tests. All experiments were done in triplicate, and the results were expressed as mean ± SD.

## Results

### Identification of *P. mirabilis* isolates

A total of 60 isolates were recovered from urine (35/60, 58.3%), followed by wounds (13/60, 21.6%), blood (7/60, 11.6%), and sputum (5/60, 8.3%).They were identified as Gram-negative rods. They produced non-lactose-fermenting colonies on MacConkey’s agar, swarming on nutrient agar, and hydrogen sulfide from Kligler’s iron agar. They were positive for urease and phenylalanine deaminase production and negative for indole production.

### Antimicrobial susceptibility patterns

*P. mirabilis* isolates (n = 60) showed variable degrees of resistance pattern to the representative antibiotics used. The percentages of antimicrobial resistance were as follows: gentamycin (40%), tobramycin (13.3%), piperacillin + taobzactam (28.3%), imipenem (0%), cefazolin (83.3%), cefotaxime (93.3%), cefepime (11.7%), cefoxitin (4%), ciprofloxacin (76.7%), trimethoprim-sulfamethoxazole (93.3%), Aztreonam (93.3%), ampicillin (85%), amoxicillin + lavulanic acid (55%), doxycycline (78.3%).

### Phenotypic determination of virulence factors

The phenotypic characterization of the virulence factors showed that 45 out of 60 isolates (75%) had the capacity to produce biofilm, including 11 isolates (18.3%), which were strong biofilm producers, 28 isolates (46.6%), which were moderate biofilm producers, and the rest (6 isolates) (10%), which were weak producers. All the isolates (n = 60) showed motility and were positive for urease production, while 10 and 8 isolates (16.6% and 13.3%) showed positivity for protease and hemolysin production, respectively. The positive isolates (n = 8) for all virulence determinants and strong biofilm producers were selected for further investigations.

### MIC determination

The MICs of CIP against *P. mirabilis* isolates (n = 8) were determined according to CLSI guidelines, and the results obtained are presented in Table 2. The MIC values ranged from 2 to 16 μg/ml.

**Table 2:** MIC values for *P. mirabilis* isolates against ciprofloxacin

Isolate code	MIC value (μg/ml)	Isolate code	MIC value (μg/ml)
P1	8	P18	4
P10	16	P38	16
P16	16	P39	16
P17	2	P55	16

### Growth curve assay

**Fig. 1:**
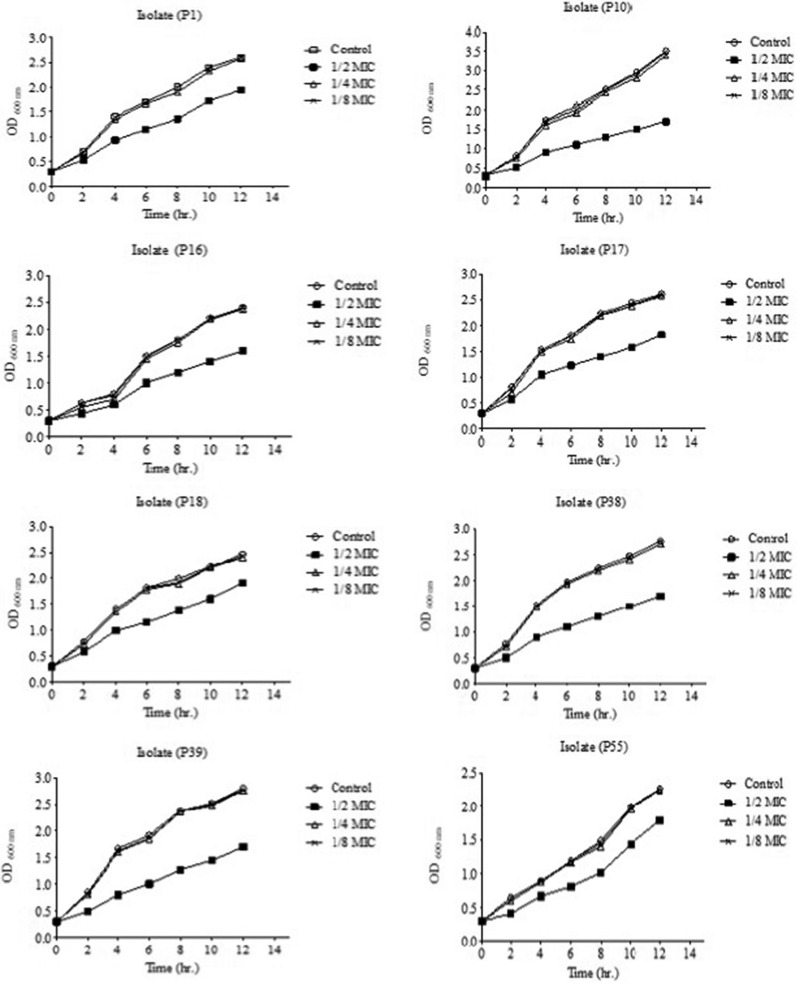
Growth curve of *P. mirabilis* isolates that were grown in the absence and presence of 1/2, 1/4, and 1/8 MICs of ciprofloxacin. The results were the mean of three experiments

The growth of *P. mirabilis* isolates with and without CIP (1/2, 1/4, or 1/8 MICs) was tested at OD_600_ (Fig. 1). According to the obtained growth curves, 1/2 MIC of CIP greatly affected the growth rate of the isolates, while 1/4 or 1/8 MICs of CIP had negligible effects on the growth of the isolates, and so they were selected for further experiments.

### The effect of sub-MICs of ciprofloxacin on biofilm formation

**Fig. 2:**
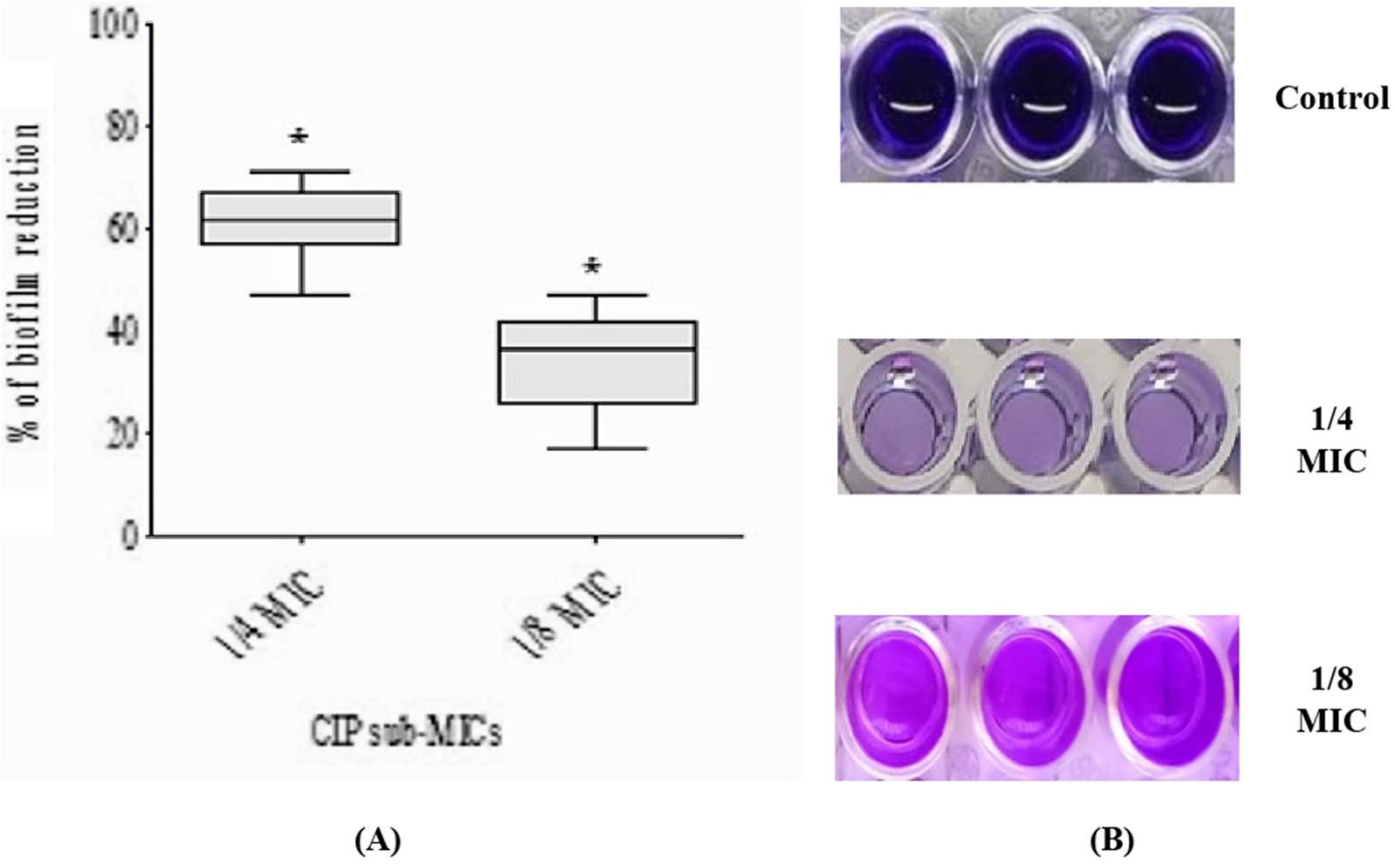
Concentration-dependent inhibition of biofilm formation of *P. mirabilis* isolates by treatment with sub-MICs of CIP using crystal violet assay. **A** The scatter plot indicates the percentage of reduction in biofilm formation after treatment with 1/4 and 1/8 MIC of CIP. **B** A representative image of crystal violet-stained biofilm of *P. mirabilis* isolate before and after treatment at different concentrations in a microtiter plate. The results were the mean of three experiments. The error bars indicate standard deviations. The asterisks represent statistical significance (p < 0.05)

CIP at sub-MICs (1/4 or 1/8 MICs) significantly (p < 0.05) reduced biofilm development in all *P. mirabilis* isolates in a concentration-dependent manner. The reduction was higher at 1/4 MIC, with percent ranging from 47.3% to 70.9%, while at 1/8 MIC, it ranged from 16.9% to 47.2% (Fig. 2).

### The effect of sub-MICs of ciprofloxacin on motility

**Fig. 3:**
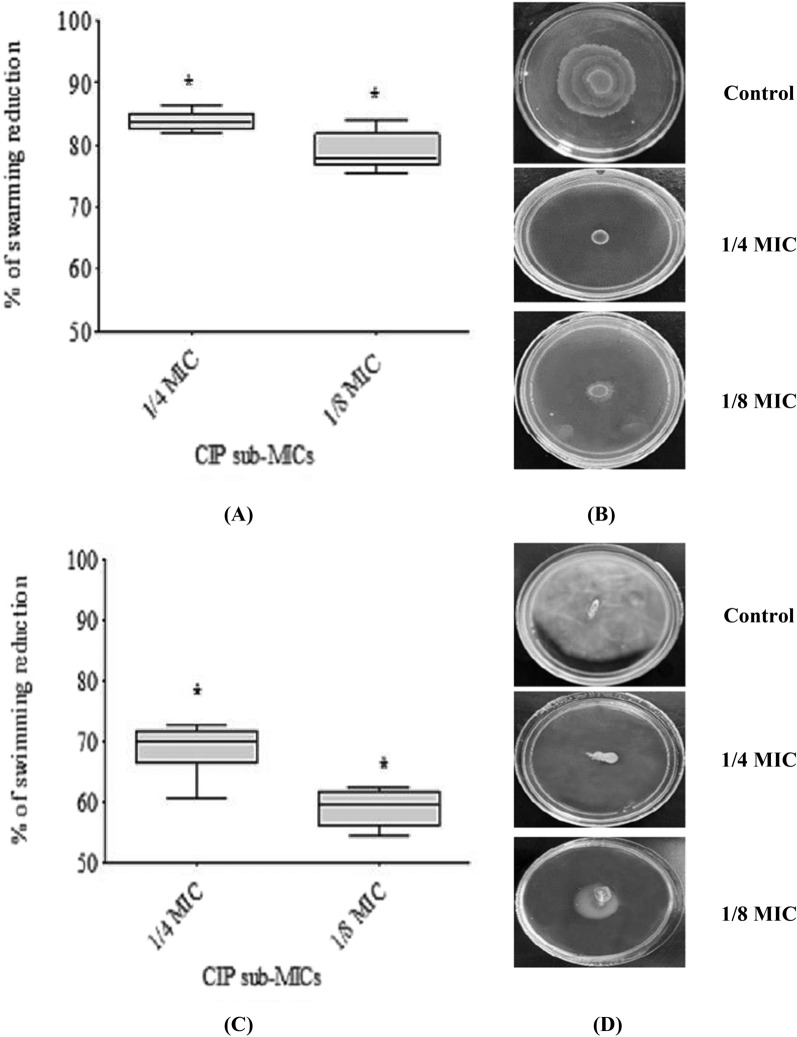
Inhibition of *P. mirabilis* motility by sub-MICs of CIP. **A**, **C** The scatter plots indicate the percentage of reduction in swarming and swimming motilities, respectively. **B** Representative images of the effect of CIP at 1/4 and 1/8 MIC on swarming motility compared to control (no CIP). **D** Representative images of the effect of CIP at 1/4 and 1/8 MIC on swimming motility compared to control (no CIP). The results were the mean of three experiments. The error bars indicate standard deviations. The asterisks represent statistical significance (p < 0.05)

The impact of CIP at 1/4 MIC or 1/8 MIC showed a significant (p < 0.05) inhibition of the swarming and swimming motilities of all *P. mirabilis* isolates while having no discernible influence on growth (Fig. 3). Swarming motility was suppressed by (81.8–86.3%) at 1/4 MIC and (75.49–84%) at 1/8 MIC, whereas swimming motility was inhibited by (60–72.8%) at 1/4 MIC and (54.3–62.4%) at 1/8 MIC.

### The effect of sub-MICs of ciprofloxacin on virulence enzymes and toxins

**Fig. 4:**
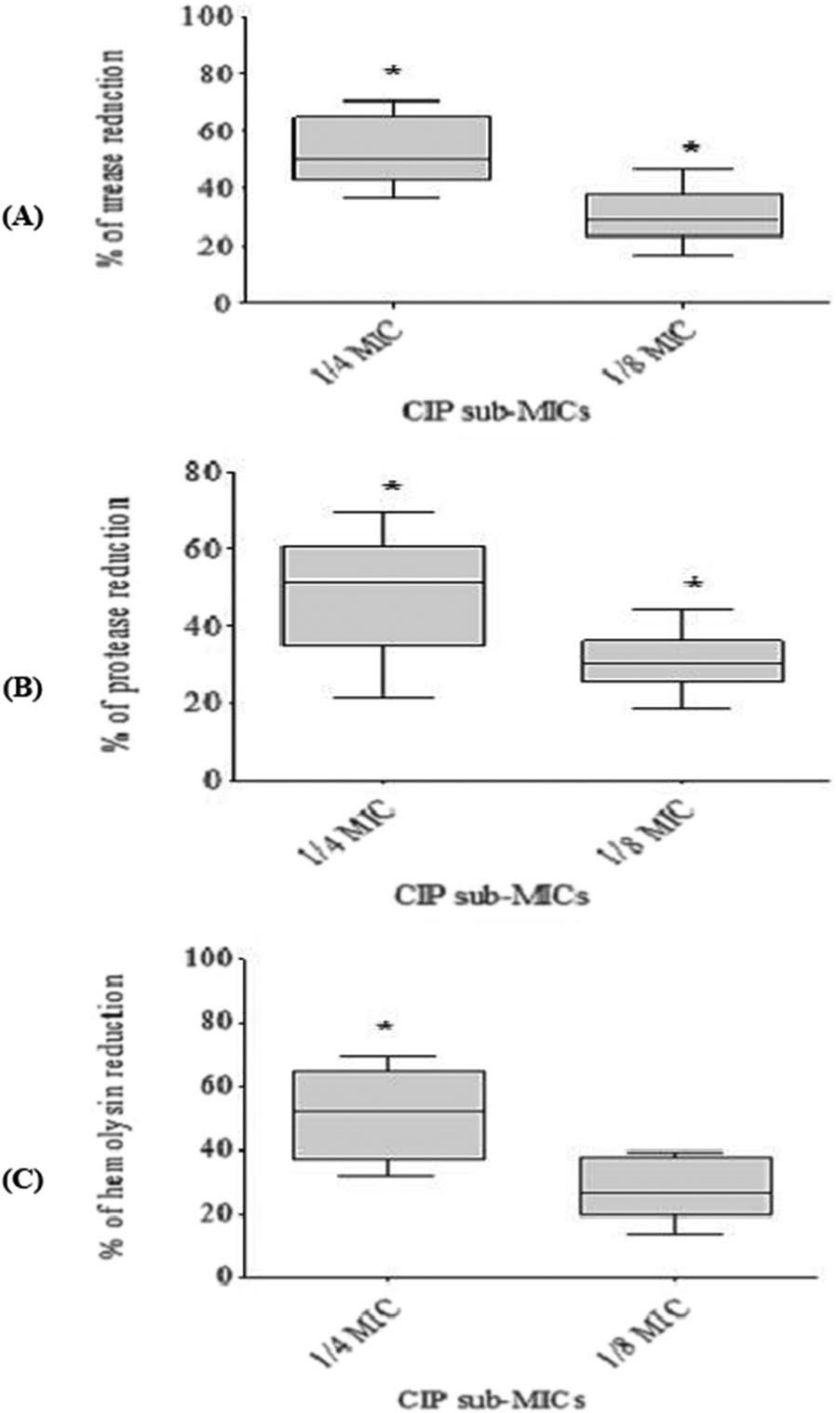
Concentration-dependent inhibition of *P. mirabilis* virulence. The scatter plots indicate the percentage of reduction in (**A**) urease, (**B**) protease, and (**C**) hemolysin production after treatment with 1/4 and 1/8 MIC of CIP. The results were the mean of three experiments. The error bars indicate standard deviations. The asterisks represent statistical significance (p < 0.05)

The production of urease, protease, and hemolysin was attenuated in all *P. mirabilis* isolates in a concentration-dependent way when treated with CIP at sub-MICs (Fig. 4). For urease production, it was significantly (p < 0.05) reduced by (36.7–70.6%) at 1/4 MIC and by (16.9–46.8%) at 1/8 MIC. For protease production, it was suppressed significantly (p < 0.05) by (21.5–69.4%) at 1/4 MIC and by (18.5–43.9%) at 1/8 MIC. For hemolysin production, it exhibited a significant (p < 0.05) reduction ranging from 31.8 to 69.9% at 1/4 MIC. However, it did not exhibit a statistically significant reduction at 1/8 MIC.

### The impact of sub-MICs of ciprofloxacin on cell morphology

**Fig. 5:**
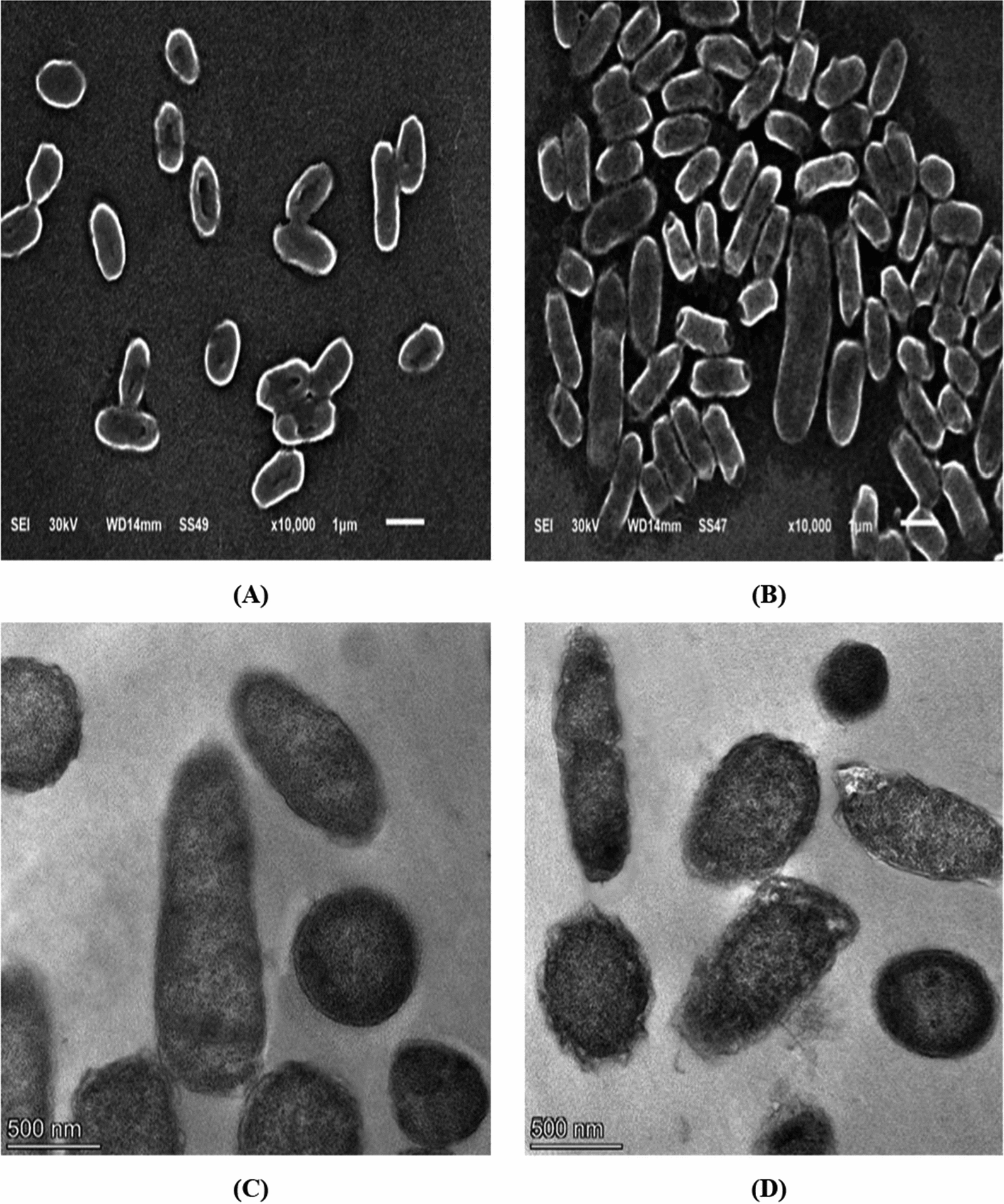
Electron microscope images of a representative *P. mirabilis* isolate (**A**) scanning image before, (**B**) scanning image after treatment with sub-MIC of CIP, (**C**) transmission before and (**D**) after treatment with sub-MIC of CIP

By exposing the representative isolate (code P17) to 1/4 MIC of CIP, there was enlargement in some cells with irregular morphology showed by SEM compared to untreated cells. Also, the TEM investigation showed that nuclear morphology changes appear as some mesosome-like structures and non-membrane-enclosed bodies are formed with changes in the cell wall and cell membrane integrity (Figs. 5).

### The impact of sub-MIC of ciprofloxacin on gene expression

**Fig. 6:**
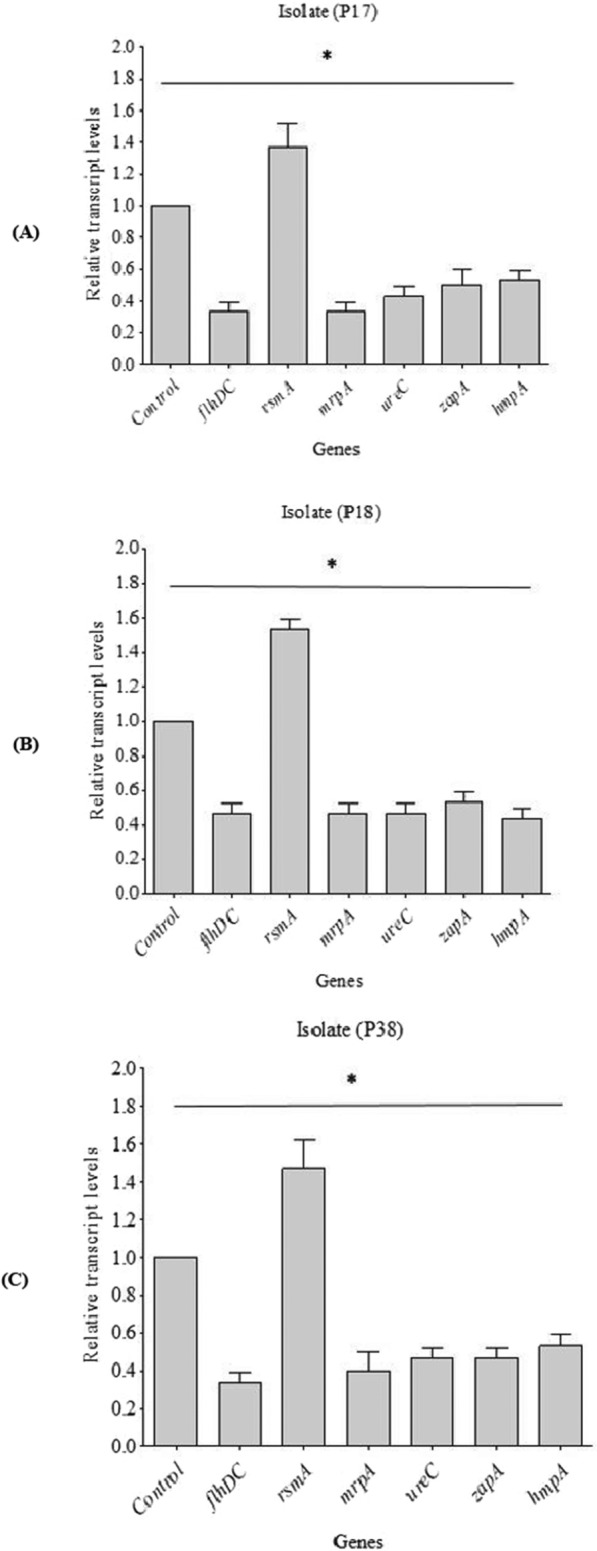
Relative transcription levels of selected genes of 3 P. mirabilis isolates (**A**) P17, (**B**) P18, and (**C**) P38 after treatment with sub-MIC of CIP. The error bars indicate standard deviations. The asterisks represent statistical significance (p < 0.05)

Quantitative real-time PCR was used to study the effect of ciprofloxacin (at 1/4 MIC) on the three representative isolates (code P17, P18, and P38) that were most affected in the previous assays. The mRNA expression levels of treated samples were calibrated relative to the control group (in the absence of CIP). The expression of genes was significantly (p < 0.05) affected in the three isolates (Fig. 6). The data showed the downregulation of *flhDC*, *mrpA*, *ureC*, *zapA*, and *hmpA* genes. It also revealed the upregulation of *rsmA*.

### Histopathological examination of wound infection

**Fig. 7:**
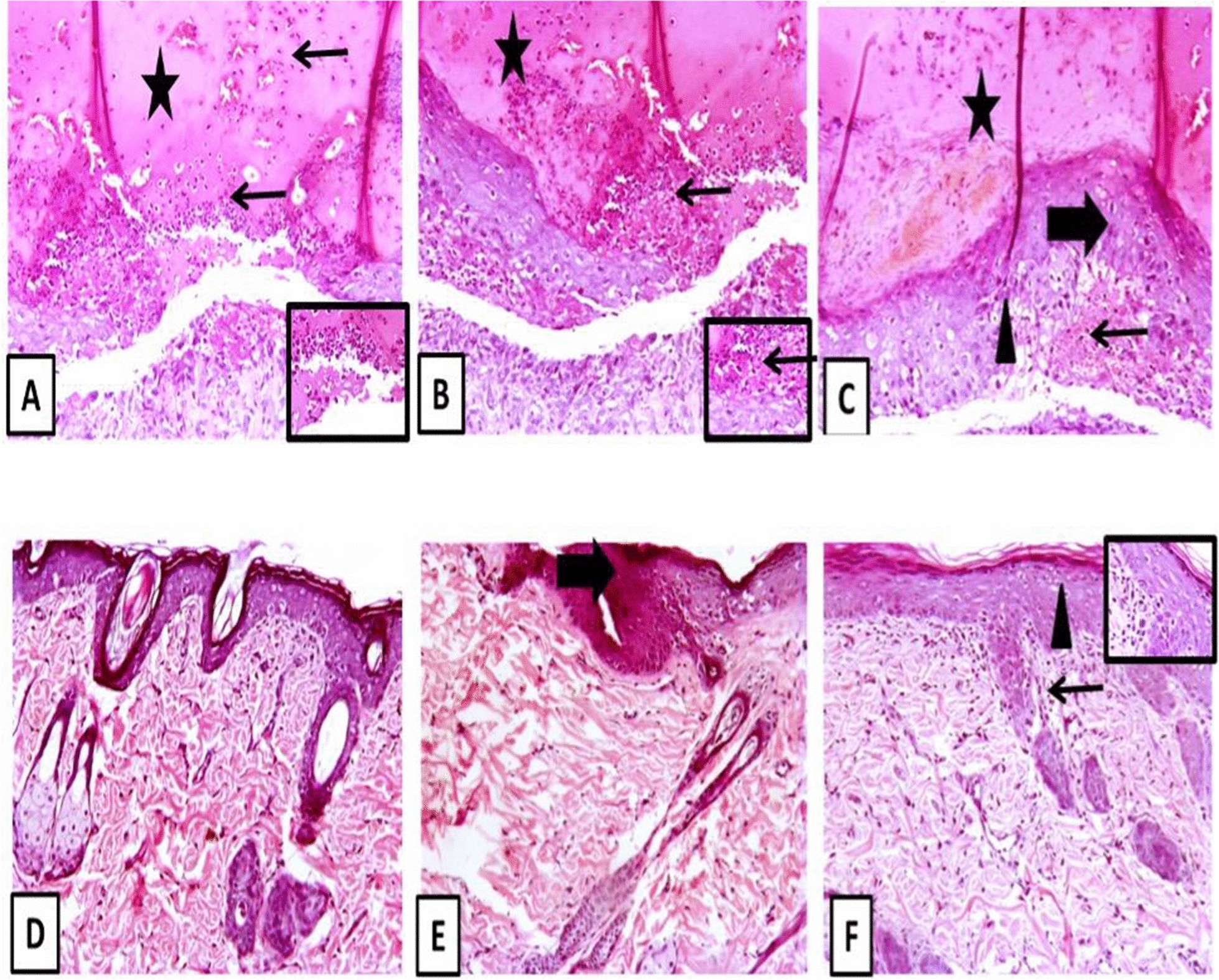
Representative photomicrograph of different treatment groups. **A**, **B** Control untreated group showing replacement of ulcertaed skin area with large esinophilic scab (stars) invaded with many inflammatory cells including many neutrophils (thin arrows). Many inflammatory cells admixed with many necrotic epidermal cells filling the wound gap. **C** Control untreated group showing closure of wound area with reepithelized epidermal cells and angiogenesis (thin arrow). Many epidermal cells showing vacuolation (arrowhead) with acanthosis (thick arrow) and epidermal layer covered with thick esinophilic crust invaded with inflammatory cells (star). **D** 1/4 MIC-treated group showing complete regeneration of epidermis and dermis with normal hair follicle and sebaceous gland. **E**, **F** 1/8 MIC-treated group showing restoration of epidermal layer with either mild acanthosis (thick arrow) or epidermal vacuolation (arrowhead) with mild dermal inflammatory cells (thin arrow), see inset image. Image magnification = 100×, inset = 400×

The wound-healing potential of sub-MICs of CIP was also evaluated using H&E stains, as shown in Fig. 7. The histological examination of tissues in the control group, which was infected with *P. mirabilis* and untreated, revealed extensive tissue damage, skin ulceration accompanied by intense inflammation (acute and chronic inflammatory cells), and the presence of necrotic debris without any epithelization. The histopathological assessment of the treated groups, infected with *P. mirabilis* and treated with sub-MICs of CIP, revealed moderate to complete epithelization accompanied by underlying granulation tissue and few inflammatory cells.

The scoring of wounds demonstrated that the application of 1/4 MIC or 1/8 MIC of CIP resulted in enhanced wound angiogenesis, proliferation of fibroblasts, activation of collagen deposition-activated hair follicles, epidermal regeneration, and a reduction in the infiltration of inflammatory cells and scab formation, as indicated in Table 3.

**Table 3:** Assessment of wounds showing that 1/4 or 1/8 MIC of CIP support wound healing

Scoring	Control untreated	1/8 MIC-treated	1/4 MIC-treated
Epidermal regeneration	+	++	+++
Inflammatory infiltrates	+++	+	–
Scab formation	+++	–	–
Angiogenesis	+	+++	+++
Granulation tissue	++	++	++
Proliferation of fibroblasts	–	++	+++
Collagen deposition	–	++	+++
Activated hair follicles	–	++	+++

–; absent, +; mild, ++; moderate, +++; severe

## Discussion

*Proteus mirabilis* is one of the most prevalent causes of nosocomial infections. It plays a crucial role in urinary tract infections (UTIs). It is third in causing complicated UTIs, following *E. coli* and *Klebsiella pneumoniae*, and second in causing catheter-associated bacteriuria, following *Providencia stuartii*, in long-term catheterized patients [31]. It is also a significant contributor to the development of wound infections and was reported as a prominent Gram-negative isolate in wound infections [32, 33]. Its dissemination is due to the presence of a variety of virulence factors, including peritrichous flagella, fimbriae, urease, protease, hemolysin, and a substantial capacity to form biofilms [31]. We focused on the fluoroquinolone ciprofloxacin which is used worldwide in both human and animal sectors, and therefore has both clinical and environmental effects.

Biofilm formation enables organisms to survive harsh environments and renders them more resistant (10–1,000 times higher) to drugs as well as the immune system of the host [34]. The process of biofilm development in *P. mirabilis* encompasses a series of interconnected processes that collectively facilitate the formation of biofilms, involving the expression of adhesive proteins (in particular MR/P fimbriae), swarming motility, and urease production [1]. In addition, the seriousness of *P. Mirabilis* infection depends on flagellar motility and other virulence determinants that could affect the pathogenesis through adherence to epithelial and catheter surfaces, stone formation, cell invasion and cytotoxicity, and histological damage, as well as immune evasion [1]. In this work, we employed CIP at sub-MIC levels that had negligible influence on growth. According to our findings, sub-MIC of CIP significantly reduced biofilm formation, and other virulence determinants in a concentration-dependent way.

These findings are consistent with the relative gene expression levels determined by quantitative real-time PCR. Sub-MIC of CIP-treated isolates demonstrated considerable down-regulation of examined genes when compared to untreated control isolates. *P. mirabilis* motility is mediated through the class 1 flagellar master regulator gene, *flhDC* (flagellar transcriptional activator) [35]. CIP at sub-MIC down-regulated the relative expression of *flhDC*. The MR/P (mannose-resistant Proteus-like) fimbria is one of the most well-studied and important adherence structures produced by *P. mirabilis* [36]. Our findings showed the down-regulation of *mrpA* encoding the main pilin subunit. In addition, our findings showed down-regulation of the genes encoding other virulence, as follows: *ureC* (encoding major urease structural subunit), *zapA* (encoding protease enzyme), and *hmpA* (encoding hemolysin toxin) when exposed to CIP at sub-MIC.

Furthermore, our molecular data showed the up-regulation of the gene *rsmA*, a repressor of secondary metabolites, which is an important part of a global regulatory system controlling the expression of many genes in the stationary phase. Increased expression of *rsmA* in *P. mirabilis* inhibits swarming, differentiation of swarmer cells, and the expression of virulence factors, including hemolysin, protease, urease, and flagellin [37], as well as biofilm formation [26, 37].

Considering the observed down-regulation of *flhDC*, *mrpA*, *ureC*, *zapA*, and *hmpA*, along with the up-regulation of *rsmA*, besides the conducted phenotypic studies, it can be understood why the sub-MICs of ciprofloxacin attenuated virulence factors and biofilm development. A noteworthy previous study [38] has established a correlation between swarming behaviour and the expression of virulence characteristics, specifically invasion and the production of hemolysin, urease, and protease. Hence, the inhibition of swarming motility and the down-regulation of flagellar transcriptional activator could be associated with the inhibition and down-regulation of virulence factors.

In agreement with our study, Wojnicz et al*.* revealed that sub-MICs of ciprofloxacin showed inhibition of adherence and attachment capabilities to epithelial cells [39]. Additionally, Drago et al*.* documented that quinolones at sub-MICs exert anti-adherence activity [40]. Also, Abdullah et al*.* reported that ciprofloxacin at sub-MICs exerts anti-urease activity on *P. mirabilis*, which is a cornerstone in pathogenicity and biofilm formation [41]. Furthermore, Gupta et al*.* proved that exposure to a sub-MIC of ciprofloxacin inhibits the production and expression of virulence like protease and hemolysin [42]. In addition, Dong et al*.* and Gümüş et al*.* demonstrated that ciprofloxacin at sub-MICs reduced the relative expression levels of virulence genes [17, 43]. Horii et al*.* reported that the sub-MIC of mupirocin dose-dependently suppressed bacterial motility and flagella formation in *P. mirabilis* with reduced flagellin expression [44]. As well, Kawamura-Sato showed, based on molecular analysis, that the inhibition of motility by sub-MICs of macrolides in *P. mirabilis* was well correlated with reduced expression of flagellin [45]. Besides, Roudashti et al*.* showed that the sub-MICs of ciprofloxacin significantly reduced motility expression and biofilm formation [46]. Over and above that, there are previous studies that reported a significant reduction in the biofilm formation of *P. mirabilis* when exposed to sub-MICs of ciprofloxacin [47–49].

Moreover, bacteria could adapt to stress in their environment by altering their morphology and ultrastructure. We studied the effect of CIP at sub-MIC using SEM and TEM, which revealed changes in cell size, shape, and cell wall integrity. These findings agree with a previous study by Zhanel et al. [50]. Also, Kwon and Lee showed that exposure to sub-MICs has been shown to induce changes in cell morphology, which may directly interfere with the expression of virulence [51].

Finally, we investigated the impact of CIP at 1/4 or 1/8 MIC on the process of wound healing in mice. The findings of our study revealed that CIP had a positive influence on promoting wound healing. This was determined by histopathological examination, which also demonstrates an increase in epidermal regeneration, collagen deposition, granulation tissue creation, and hair follicles while simultaneously reducing inflammation. Zhanel et al. also observed that subinhibitory concentrations of antimicrobials resulted in reduced bacterial counts, lower histological injury, and longer survival rates compared to the control group [50]. Our findings align with studies that concluded that subinhibitory concentrations of antimicrobials have protective effects in animal models, possibly involving the modulation of virulence factors, a reduction in bacterial adhesion, and decreased infectivity [50].

## Conclusion

The application of ciprofloxacin at sub-minimal inhibitory concentrations (sub-MICs), which exhibited noteworthy concentration-dependent inhibitory effects on the formation of biofilms in vitro, as well as the reduction of virulence factors such as motility, enzymes, and toxin production. Our findings illustrated the significance of selecting antibiotics with care and caution, taking their concentrations seriously into consideration, particularly ciprofloxacin, in order to treat *P. mirabilis* infections and enhance patient outcomes.

## Data Availability

All data generated or analyzed during this study are included in this article.
